# The Effect of Low-Level Laser on Postoperative Pain After Tibial Fracture Surgery: A Double-Blind Controlled Randomized Clinical Trial

**DOI:** 10.5812/aapm.17350

**Published:** 2014-06-21

**Authors:** Sholeh Nesioonpour, Soheila Mokmeli, Salman Vojdani, Ahmadreza Mohtadi, Reza Akhondzadeh, Kaveh Behaeen, Shahnam Moosavi, Sarah Hojjati

**Affiliations:** 1 Department of Anesthesiology, Pain Research Center, Ahvaz Jundishapur University of Medical Sciences, Ahvaz, Iran; 2 Canadian Optic and Laser Center, COL Center, Victoria, Canada; 3 Department of Orthopedic, Ahvaz Jundishapur University of Medical Sciences, Ahvaz, Iran; 4 Department of Physical Education and Sport Science, Bu-Ali Sina University, Hamedan, Iran

**Keywords:** Low Level Laser Therapy, Postoperative Pain, Tibial Fracture Surgery

## Abstract

**Background:**

Postoperative pain is a common complication that can lead to serious morbidities and delayed recovery.

**Objectives:**

The aim of this study was to investigate the effect of low-level laser therapy on acute pain after tibial fracture surgery.

**Patients and Methods:**

In this randomized clinical trial, 54 patients who were candidate for tibial fracture surgery were allocated randomly to two groups, namely, control and laser therapy. Both groups had the same type of surgery and technique of spinal anesthesia. Patients in laser group were treated with the combination of two lasers (GaALAs, 808 nm; and GaALInP, 650 nm) at the end of the surgery while control group received laser in turn-off mode with the same duration as laser group. Patients were evaluated for pain intensity according to the visual analogue scale (VAS) and the amount of analgesic use during 24 hours after surgery.

**Results:**

Laser group experienced less pain intensity in comparison with control group at second, fourth, eighth, 12^th^, and 24^th^ hours after surgery (P Value < 0.05). In addition, the amount of consumed opioid in laser group was significantly less than the control group (51.62 ± 29.52 and 89.28 ± 35.54 mg, respectively; P Value, 0.008).

**Conclusions:**

Low Level Laser Therapy is a proper method to reduce postoperative pain because it is painless, safe, and noninvasive and is easily accepted by patients.

## 1. Background

One of the undesirable complications of surgery is postoperative pain that may result in serious morbidities such as agitation, hypertension, mood changing, tachycardia (1, 2) and delay in wound healing, which can be more dangerous in patients with the underlying diabetes mellitus, hypertension, or coronary heart diseases as it may lead to fatal complications such as myocardial infarction (3). There is a high variability among patients in tolerance to pain and analgesic requirement (4, 5). The studies show that about 80% of patients experience a mild to severe pain after surgery (6). There is inadequate postoperative analgesia in the half of all surgeries, can lead to chronic postoperative pain (7). Several methods are available to control and reduce postoperative pain such as administering opioids or nonsteroidal anti-inflammatory drugs (NSAIDs) and patient-controlled analgesia (PCA). It is established that the use of systemic opioids alone is not sufficient to relieve postoperative pain. In most cases, inadequate dosage is prescribed to reduce the side effects of these drugs like respiratory depression and therefore, the medication cannot control pain completely (8, 9). Analgesic nephropathy, skin reactions, and peptic ulcers are common side effects of nonsteroidal anti-inflammatory drugs (10). Recent advances present new techniques for prevention and reduction of postoperative pain. One of the most important technologies of this century is the use of low-level laser (LLL) at the site of surgery (11).

Low-level laser therapy (LLLT) was pioneered at Russia and Hungry and then at Europe in early 1960s. It is a branch of laser treatments that has been indicated for pain killing and wound healing. LLLT uses irradiation with laser light of low intensity and its effects are not due to producing heat. These nonthermal effects are thought to be mediated by a photochemical reaction that alters cell membrane permeability, leading to increased mRNA synthesis and cell proliferation. FDA has started different investigations on LLLT for 15 years and has approved the use of LLLT for pain relief in carpal tunnel syndrome since 2002 (11, 12). It is also used to treat damages in sport injuries and musculoskeletal disorders. In addition, it is applicable to reduce neck pain and the size of keloid scarring after surgery (13-17). Many studies found that LLL stimulates respiratory cycle in mitochondria and increases adenosine triphosphate molecules (14) that reduce swelling and pain (16). In another study, applying LLL directly over painful points was useful in treatment of stress fracture of tibia (18). The LLL is effective in relieving pain of knee osteoarthritis, breast augmentation surgery, and cryosurgical treatment of oral leukoplakia (15, 17).

## 2. Objectives

Pain following orthopedic surgeries are considered severe pain (19, 20); hence, the aim of this study was to investigate the effect of LLLT on acute pain after tibial fracture surgery.

## 3. Patients and Methods

This double-blind, controlled, randomized clinical trial was conducted in 2012-2013 in Imam Khomeini Hospital, Ahvaz, Iran. The study was approved by the Ethical Committee of Jundishapur University of Medical Sciences (ETH-654) and all subjects signed an informed consent.

Sample size was calculated at 27 in each arm of the study by setting the power at 80% and the values for Z_1-α/2_, Z_1-β_, P_1_, and P_2_ at 1.96, 0.84, 0.68, and 0.32, respectively, based on a previous observational study (21). A total of 54 patients aged between 18 and 60 years who were candidate for tibial fracture surgery in American Society of Anesthesiologists (ASA) classes I and II were allocated randomly to two equal groups of control and laser. All subjects were matched based on their age, weight, and height. Patients who were pregnant, those with malignant tumors, benign tumors with malignant potential, hypersensitivity to light, e.g. systemic lupus erythematosus, coagulopathies, high intracranial pressure, history of chronic pain, those on long-term opioids or other painkillers during the preceding month, or those who did not agree to undergo spinal anesthesia were excluded from the study.

Monitoring equipment including electrocardiograph, pulse oximeter and sphygmomanometer were employed for all patients; they received 10-mL/kg intravenous lactated Ringers’ solution and then spinal anesthesia was induced by the anesthesiologist.

Spinal anesthesia was induced by intrathecal administration of 10-mg 0.5% bupivacaine (Astrazeneca Co., Germany) with 25-gauge needle in the sitting position and with the midline technique.

If the systolic blood pressure dropped by 20% or more, 10-mg ephedrine would be injected intravenously. Upon achieving successful anesthesia, pull-tight elasticated tourniquet was clamped and operation was started. The surgical procedures were similar in both groups and included open reamed interlocking intramedullary nailing, which is the preferred approach for treatment of tibial shaft fractures (22). 

After the surgery and before the final bandage in surgery room, patients in laser group were treated with a combination of two lasers (Canadian Optic and Laser Center, Canada):

1. GaALAs hand held probe (PLP-IR) with wavelength of 808 nm and 300-mW output power in continuous mode (dose, 6 J/cm^2^; area, 1 cm^2^; and time, 20 s/point); and
2. GaALInP hand held probe (PLP-R) with wavelength of 650 nm and 100-mW output power in continuous mode, (dose, 3 J/cm^2^; area, 1 cm^2^; and time, 30 s/point).

Each tibial fracture was radiated from four sides in contact technique with the combination of IR and R laser in dose of 9 J/cm^2^ (medial, lateral, anterior, and posterior sides of fracture region and popliteal fossa). For radiation on popliteal fossa, the legs were elevated by 60° angels.

In addition, trigger points on muscles and surgical wounds (6-8 points) were radiated with 4 J/cm^2^ by the same combination of IR and R lasers (ten seconds of each laser; 3 J/point IR plus 1 J/point R laser).

For placebo laser treatment in control group, all those sites were treated with the lasers in turn-off mode with the same duration.

One of authors who was blind to the group allocation and did not participate in the laser therapy procedures, filled out the questionnaires. The amount of total analgesic and pain intensity at second, fourth, eighth, 12^th^, and 24^th^ hours after the surgery were investigated in both groups. Pain intensity was quantified by visual analogue scale (VAS) in which zero and ten represented analgesia and worst possible perception of pain, respectively. If VAS was three or more, 0.3 mg/kg of pethidine was injected intravenously.

### 3.1. Statistical Analysis

The data are presented as mean ± standard deviation (SD). We performed Shapiro-Wilk test and Levene's test for normality of the data distribution and equality of variances. Independent samples t test, repeated measure test, and Bonferroni post hoc test were used to analyze the data. P Value of less than 0.05 was considered as statistically significant. All the statistical analyses were done by SPSS software version 16 (SPSS Inc, Chicago, IL, USA).

## 4. Results

Demographic characteristics of participants are presented in Table 1. Two groups were similar in terms of age, weight, height, and body mass index. There was no significant difference between groups regarding the duration of surgery (57.34 ± 3.2 and 56.29 ± 3.4 minutes in control and laser groups, respectively; P = 0.71) and anesthesia duration (84.14 ± 5.21 and 85.02 ± 4.98 minutes in control and laser groups, respectively; P = 0.69).

**Table 1.** Demographic Characteristics of the Participants^a,b^

Groups	Age, y	Weight, kg	Height, cm	BMI, kg/m^2^
**Control Group**	24.61 ± 2.76	71.22 ± 11.34	169 ± 6	72.16 ± 12.71
**LLLT Group**	25.05 ± 2.68	72.27 ± 10.80	171 ± 5	70.09 ± 13.23
**P value**	0.628	0.777	0.791	0.706

^a^Abbreviations: LLLT, low-level laser therapy; and BMI, body mass index.

^b^Data are presented as mean ± SD.

**Figure 1.**
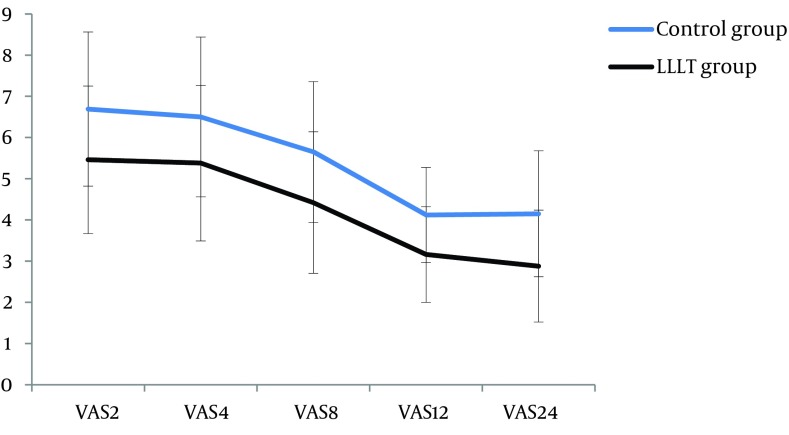
Pain Intensity at Different Hours in lllt and Control Groups

Based on VAS, mean scores of pain intensity after operation in different periods are presented in Table 2. Pain reduced considerably at second, fourth, eighth, 12^th^, and 24^th^ hours after surgery in laser group in comparison with the control group. Although there were no significant differences in pain intensity between the second and fourth, the fourth and eighth, the eighth and 12^th^, as well as the 12^th^ and 24^th^ hours in each group (P > 0.999, P = 0.110, P = 0.681, and P > 0.999 in control group; P > 0.999, P = 0.099, P = 0.097, and P > 0.999 in laser group, respectively), there were significant differences between the second and eighth, the second and 12^th^, the second and 24^th^, the fourth and 12^th^, the fourth and 24^th^, as well as the eighth and 24^th^ in each group (P < 0.001, P = 0.010, P < 0.001, P = 0.009, P < 0.001, and P = 0.002 in control group; P < 0.001, P = 0.002, P < 0.001, P = 0.002, P < 0.001, and P < 0.001 in lase group, respectively; Figure 1).

The mean of total amount of analgesic (pethidine) used in laser group was significantly less than control group. The mean of total amount of analgesic was 51.62 ± 29.52 and 89.28 ± 35.54 mg in laser and control groups, respectively (P = 0.008).

**Table 2.** Postoperative Pain Intensity^a, b^

Groups	VAS at Different Time Points After Surgery				
	2^nd^ h	4^th^ h	8^th^ h	12^th^ h	24^th^ h
**Control Group**	6.69 ± 1.87	6.50 ± 1.94	5.65 ± 1.71	4.12 ± 1.15	4.15 ± 1.53
**LLLT Group**	5.46 ± 1.79	5.38 ± 1.89	4.42 ± 1.72	3.16 ± 1.16	2.88 ± 1.36
**P value**	0.019	0.041	0.013	0.006	0.012

^a^ Abbreviations: LLLT, low-level laser therapy; and VAS, visual analogue scale.

^b^ Data are presented as mean ± SD.

## 5. Discussion

Pain as a stressor, stimulates the physiological and psychological responses. Its outcomes have a direct effect on the postoperative complications, recovery time, and patient’s satisfaction with the health system. The aim of this study was to investigate the effect of LLL with the wavelengths 650 and 808 nm on pain after tibial fracture surgery. The results of this study showed that pain reduction was significant at the second, fourth, eighth, 12^th^, and 24^th^ hours after surgery (P Value ≤ 0.05). Similarly, Moore et al. showed that low level gallium-aluminum-arsenide laser for four to six minutes at the end of the cholecystectomy had no significant effect on pain reduction at the first and the fourth hours after surgery; however, the effect was significant at the eighth, 12^th^, 24^th^, and 48^th^ hours after surgery (21). Hegedus et al. reported that the use of LLL (wavelength, 830 nm; continuous wave; and power, 50 mW) in patients with knee osteoarthritis resulted in pain reduction and improvement in joint movement (15). Jackson et al. found that laser irradiation with wavelength of 630 to 640 nm at the beginning and at the end of breast augmentation surgery reduced the postoperative pain (23). Moreover, Ribeiro et al. reported that AsGaAl laser (660 nm) could decrease the pain as well as postoperative recurrence rate in patients with oral leukoplakia (17). 

The results of our study showed the mean total amount of analgesic use in laser group was significantly lower than the control group (P < 0.05). This finding is consistent with the findings of other researchers who reported that LLLT could decrease pain during and after the surgery and had a positive effect on wound healing and edema (12). LLLT is used in muscular fatigue (24), wound healing, and pain reduction in dental procedures in patients with and without diabetes (25-27). The researches showed that LLL could cause analgesia by reducing prostaglandin E2 (28, 29), raising endorphin level, and increasing urinary excretion of serotonin, the pain receptors stimuli. LLLT also has a negative effect on pain neurotransmitters and prevents accumulation of acetylcholine, a pain stimulus in the receptors (30).

The results of this study showed that the combination of laser therapy and analgesic medications had better effect during the 24 hours of recovery after the surgery. Laser radiation at wavelengths of 650 and 808 nm (R and IR laser) can decrease postoperative pain and analgesic use in postoperative period. LLLT does not have side effects like respiratory depression, skin reaction, and analgesic nephropathy that are seen with other methods. It is recommended to perform more studies concerning the applications of LLLT in anesthesia field as it is a noninvasive, safe, and acceptable analgesic method in patients in recovery or surgery room.
